# Dental unit waterline testing practices: an 11-Year retrospective study

**DOI:** 10.1186/s12903-023-03590-y

**Published:** 2023-11-15

**Authors:** Juan M. Buitrago, Rob J. Kolbe, Michelle F. Siqueira

**Affiliations:** College of Dentistry, University of Saskatchewan, Saskatoon, SK Canada

**Keywords:** Biofilm(s), Clinical practice guidelines, Dental public health, Evidence-based dentistry/health care, Infection control, Microbiology

## Abstract

**Objectives:**

This retrospective study examined the dental unit waterline (DUWL) testing practices of Saskatchewan dental clinics over a period of 11 years, with an emphasis on their responses after identification of high microbial levels.

**Materials and methods:**

Dental clinics (n = 137) aseptically collected samples of output water from their air/water syringes, handpieces, and ultrasonic scaler lines using Sigma-Aldrich® waterline test kits and delivered them to a quality assurance laboratory. Tests were incubated for seven days at room temperature, and those with heterotrophic plate counts > 500 CFU/mL were reported as failures. Statistical analyses were performed on a database containing 4,093 test results.

**Results:**

Participating clinics submitted an average of 11 DUWL tests per year. Overall, 21% of tests failed, and a moderate positive association (*r*_*s*_*=.*52, *p* < 0.001) was found between clinics’ DUWL testing frequency and failure rate. Only 7% of failed DUWL tests were followed up by collection of a subsequent test within two weeks, of which 47% still exceeded the 500 CFU/mL threshold.

**Conclusions:**

Our findings demonstrate an association between DUWL testing frequency and detection of unacceptable microbial levels, along with infrequent retesting and often-inadequate intervention after a failed test. This suggests the need for further efforts at the regulatory and educational levels to maintain adequate water quality during dental treatment.

**Clinical relevance:**

Procedural water can become contaminated in DUWLs and endanger patients. Regular DUWL monitoring and evidence-based interventions to treat contaminated systems are necessary to safeguard patient health.

## Introduction

Dental unit waterlines (DUWLs) are systems of narrow-bore plastic tubing that provide water to cool and irrigate oral tissues [1, 2]. They present ideal growth conditions for bacteria [1] and may represent an infection risk for both dental personnel and patients [3–6]. Although epidemiologic research on the topic is limited, case reports have linked infections with organisms such as *Pseudomonas aeruginosa*, *Legionella pneumophila*, and non-tuberculous mycobacteria to contaminated DUWLs [2, 3, 7–11]. Fatal cases of legionellosis have also been presumed to originate in the dental setting [2, 8, 12]. Other microorganisms consistently found in dental unit output water include aerobic gram-negative bacteria, fungi, and protozoa [13–16]. Contamination of DUWLs with blood-borne viruses is also thought to present a health risk, especially to immunocompromised, elderly, and chronically ill individuals [2, 13, 17]. In accordance with infection prevention and control (IPAC) best practices, ethical principles, and legal obligations, proactive steps must be taken to minimize the microbial content of procedural water.

Factors affecting the bacterial concentration in dental unit output water include the origin of the water, saliva retraction by handpieces during treatment, water stagnation, dead legs, and the use of water heaters [13, 18–20]. DUWL contamination begins when bacterial microcolonies attach to the periphery of DUWL lumen where water flow is negligible. Over time, these colonies mature into polymicrobial biofilms with enhanced tolerance to chemical germicides and host immune defenses [13, 21–24]. As water flows through DUWLs during treatment, biofilms fragment into free-floating colonies and planktonic organisms [25], gaining access to host tissues via ingestion, contact with open wounds, or inhalation of aerosolized particles [21, 26].

To prevent biofilm formation in DUWLs and reduce the microbial load in procedural water, regulatory authorities uphold that DUWLs should be purged at the beginning of each clinic day for at least two minutes and flushed for 20–30 s after each appointment [2, 22, 27, 28]. Dental units with independent water reservoirs should also be managed with daily application of disinfectant tablets and performance of shock treatments as needed [29, 30]. Because output water directly contacts the oral cavity, non-surgical procedures must comply with the national drinking water standard of < 500 colony-forming units per milliliter (CFU/mL) of heterotrophic bacteria [19, 31]. The Centers for Disease Control and Prevention (CDC) and Organization for Safety, Asepsis, and Prevention (OSAP) uphold this same standard. However, heterotrophic plate counts regard innocuous and pathogenic organisms equally. As such, it is difficult to establish a microbial concentration other than zero that can ensure a negligible risk of adverse health effects. Procedures involving open vascular sites or cutting of bone, therefore, must only include sterile solutions administered via a sterilizable or single-use syringe [27, 32]. In light of recent DUWL-associated outbreaks, sterile water has also been recommended for some non-surgical procedures such as primary tooth pulpotomy [9, 10].

Frequent DUWL testing and maintenance are thought to protect patients and dental team members from nosocomial infections [19, 31], and adherence to IPAC standards can foster public confidence in the dental profession. Such tests may be performed in-house or through external laboratory services. In the province of Saskatchewan, Canada, quarterly testing of all DUWLs is recommended [33], and annual testing via external laboratory became mandatory in 2019 [27]. However, regional DUWL testing regulations are diverse [18, 27], and reported levels of bacterial contamination vary greatly [13]. Furthermore, many jurisdictions such as Saskatchewan do not dictate specific interventions or require re-testing after discovery of contaminated water. Also, DUWL testing frequencies and retesting norms around the world are underreported. Thus, this study aims to assess the following: (i) the frequency of external laboratory DUWL testing in a sample of Saskatchewan dental clinics; (ii) the microbial concentration in their procedural water; and (iii) the rate and timing of retesting upon notification of a failed test.

## Materials and methods

### Water sampling & heterotrophic plate count

A database of DUWL tests conducted by an external quality assurance laboratory from January 1, 2011 to December 31, 2022 was accessed from the Sterilizer and Waterline Monitoring Services Lab at the University of Saskatchewan. A Letter of Exemption from the Behavioural Research Ethics Board, University of Saskatchewan, was obtained prior to data analysis. The registry comprised samples from 137 Saskatchewan dental clinics, including private practices, public health clinics, and academic centers. Each participating clinic received a set of Sigma-Aldrich^®^ HPC Total Count Sampler test kits by courier. These kits each included a collection tube and filter. Clinicians were asked to flush DUWLs for approximately 30 s, aseptically open the test, separate the filter from the collection tube, add equal amounts of water originating from their air/water syringes, handpieces, and ultrasonic scaler lines, and close the test by attaching the filter back onto the collection tube. Then, they were asked to incubate the sample for 30 s, dispose of the water, close the test, and deliver it to the Sterilizer and Waterline Monitoring Services Lab by courier.

Upon arrival at the laboratory, each test kit was incubated for seven days at room temperature in accordance with manufacturer instructions [34]. The concentration of viable planktonic organisms was estimated by regarding bacterial colonies as individual organisms. Comparison charts representing ≤ 100 CFU/mL, 101–300 CFU/mL, 301–500 CFU/mL, and > 500CFU/mL were used to approximate the bacterial density in each sample. In accordance with Canadian drinking water regulations and professional guidelines, samples exceeding 500 CFU/mL were reported as failed tests. Because DUWL treatment strategies are designed to address biofilms as a whole, no further investigations were conducted to identify specific organisms. Dental clinics were promptly notified of their results via e-mail.

### Statistical analysis

Dental clinics were assigned random numerical identifiers in Microsoft Excel 2022 to keep their identities confidential. Descriptive and inferential statistical analyses were conducted in SPSS version 28. A chi-squared goodness-of-fit test was performed to detect differences in yearly DUWL test failure rates over time. Given the presence of outliers, Spearman’s rank correlation was used to evaluate the relationship between dental clinics’ DUWL testing frequency and their DUWL test failure rate.

## Results

**Fig. 1:**
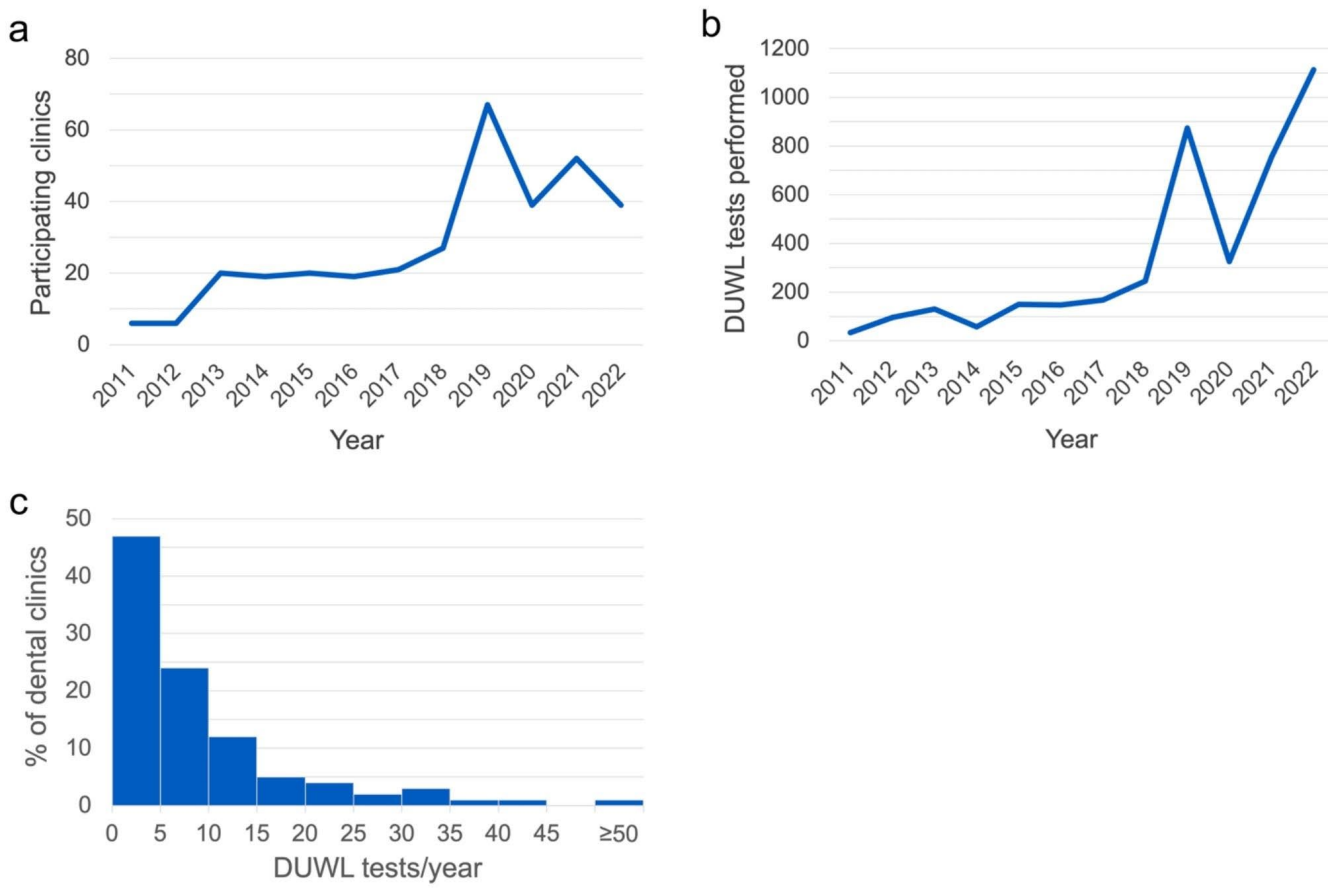
Count of **(a)** dental clinics in the Sterilizer and Waterline Monitoring Services Lab registry and **(b)** DUWL tests performed each calendar year from 2011–2022. **(c)** Histogram outlining the yearly frequency of external laboratory DUWL testing among 137 participating clinics

A total of 4,097 DUWL tests from 137 Saskatchewan dental clinics were submitted and analyzed from 2011 to 2022. The number of participating clinics and count of submitted DUWL tests generally increased over time (Fig. 1a,b). In the final year of this study, approximately 11% of all clinics registered in Saskatchewan provided DUWL tests. The mean and median testing frequencies per dental clinic were 11 and 6 tests per year, respectively (SD = 22 tests per year), with 11% of clinics submitting only a single annual test (Fig. 1c). The mean turnaround time between a clinic’s submission of a DUWL test and their notification of the results was 9 days (SD = 5 days). It should be noted, however, that data on clinic notification were not recorded from 2011 to 2013.

**Fig. 2:**
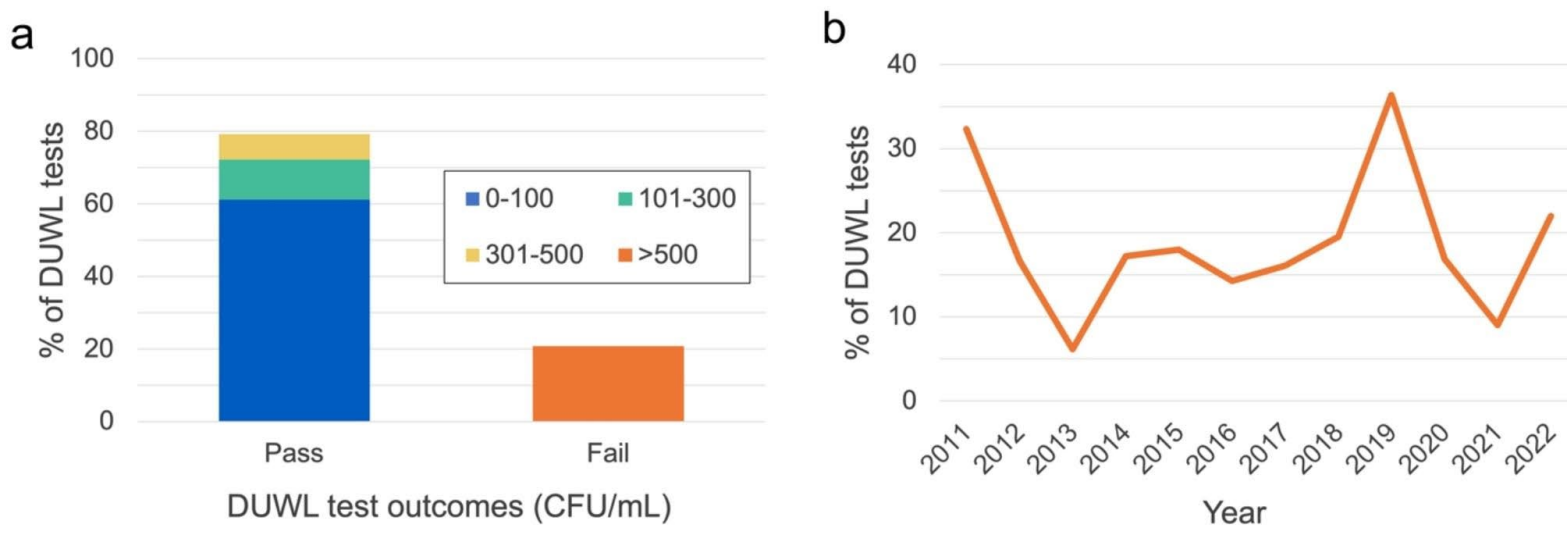
Outcomes of heterotrophic plate count DUWL tests conducted from 2011–2022. **(a)** Overall findings from 4,097 total DUWL tests. **(b)** Percentage of DUWL tests that exceeded the 500 CFU/mL threshold for safe drinking water in Canada, according to calendar year

**Fig. 3:**
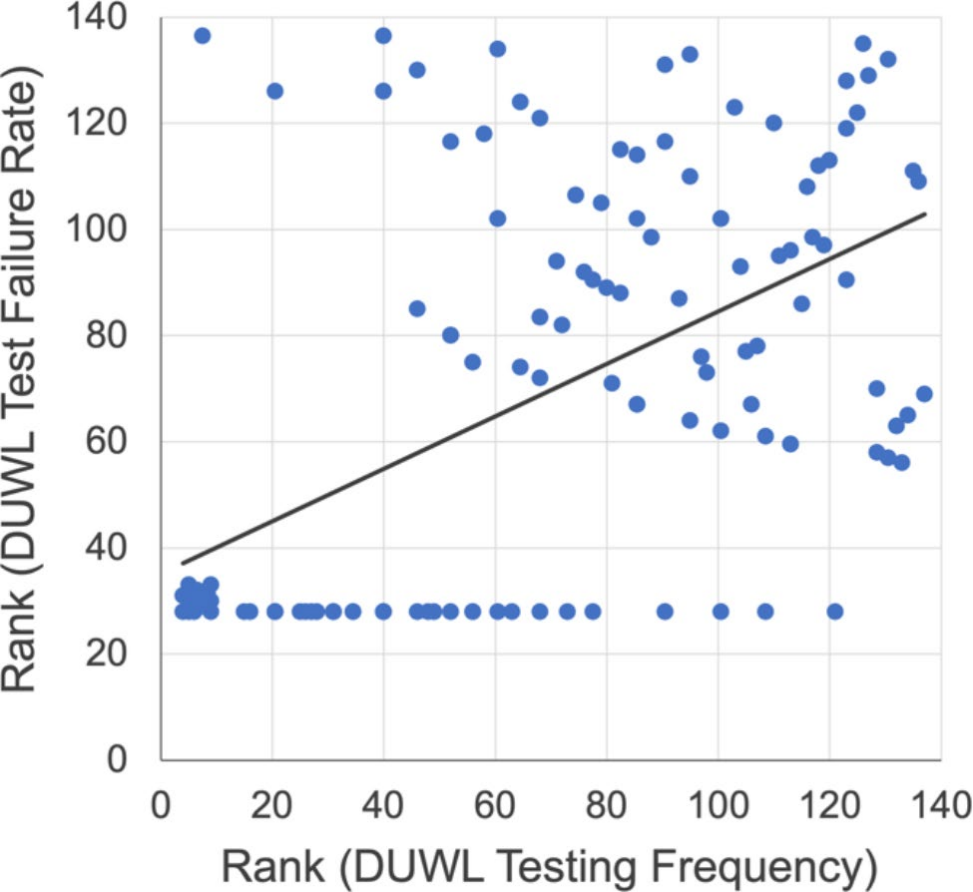
Scatter plot of ranks demonstrating a moderate positive association (*r*_*s*_*=.*52, *p* < 0.001) between participating dental clinics’ yearly DUWL testing frequency and DUWL test failure rate

Somewhat concerningly, 21% of all water samples exhibited bacterial concentrations over 500 CFU/mL (Fig. 2). A chi-squared goodness-of-fit test verified that DUWL test failure rates differed significantly by year (*p* < 0.001). Out of 137 participating clinics, 82 (60%) were notified of at least one failed test during the study period. Interestingly, individual clinic failure rates varied widely from 0 to 100%, with a mean of 19% (SD = 21%). A total of 55 clinics (40%) were found to have acceptable water quality in all collected samples, whereas two clinics (1%) only amassed failed tests (≤ 4 total tests per clinic). Paradoxically, a moderate positive correlation was identified between clinics’ frequency of DUWL testing and their rate of DUWL tests exceeding 500 CFU/mL (*r*_*s*_*=.*52, *p* < 0.001; Fig. 3).

**Fig. 4:**
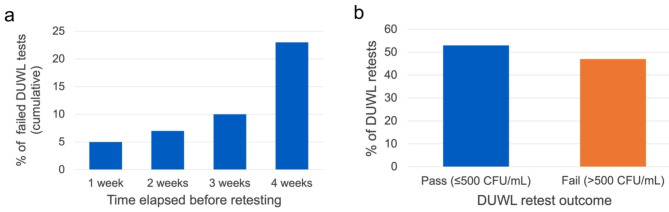
(**a)** Percentage of failed DUWL tests conducted from 2014–2022 that were followed up by collection of a subsequent sample for retesting. *Time elapsed before retesting* is defined as the interval between clinic notification and collection of a subsequent sample. ***Note*** that, even after four weeks, only 23% of contaminated DUWLs were followed up with a retest. **(b)** Outcomes of DUWL retests

Of the 852 failed DUWL tests, only 62 (7%) were followed up by collection of a subsequent sample for retesting within two weeks of clinic notification (Fig. 4a). Even after four weeks, only 23% of DUWLs with unacceptable microbial densities were retested. On average, the time elapsed between a clinic’s notification of a failed test and their submission of a subsequent sample was 106 days (SD = 178 days). Among these subsequent tests, 47% still exceeded the 500 CFU/mL standard (Fig. 4b).

## Discussion

Although procedural water is crucial for the delivery of high-quality dental care, it carries the risk of pathogen exposure and cross-infection. This retrospective study investigated DUWL testing practices among Saskatchewan dental clinics, with an emphasis on their responses after identification of high microbial levels. Indeed, 21% of all DUWL tests in this study exceeded 500 CFU/mL of heterotrophic bacteria, failing to meet Canadian drinking water standards or guidelines set by the CDC and OSAP. The lowest yearly DUWL test failure rate was observed in 2013 (6%), with the highest in 2019 (36%). Results from similar studies are highly diverse. One UK study [13] found that output water in 72% of 68 participating dental clinics exceeded 500 CFU/mL. After educating clinicians on appropriate disinfection protocols, their failure rate decreased to 13%. In China, where water standards are not enforced by regulatory authorities, bacterial concentrations as high as 1.8 × 10^6^ CFU/mL have been obtained from dental units [18, 35]. Interestingly, an Italian study found that 38% of 84 dental units with independent reservoirs exceeded the 500 CFU/mL threshold, whereas all 16 units connected to the municipal water supply satisfied the CDC guideline [7]. This heterogeneity in water quality may be related to variations in local regulations, IPAC education, and DUWL monitoring guidelines. Nevertheless, it is evident that output water from a substantial proportion of dental units around the globe does not meet safe drinking water standards.

Perhaps of greater concern are the scant responses observed in this study upon clinics’ notification of a failed DUWL test. Only 7% of water samples exceeding 500 CFU/mL were followed up within two weeks by collection of a subsequent sample for retesting. Of the subsequent tests, 47% still exhibited unacceptable bacterial concentrations. This suggests that either no interventions were performed, or that efforts to intervene did not follow evidence-based DUWL treatment protocols. While the authors recognize that in-office tests may have also been used for retesting, the outcomes of external laboratory DUWL retests in this study remain worrisome. The CDC and OSAP recommend immediate intervention for affected dental units and retesting to confirm acceptable microbial levels prior to resumption of patient care [33]. To promote such actions, further efforts at the regulatory and educational levels are encouraged.

Mandates for frequent DUWL monitoring are important administrative controls that permit timely identification of flaws in disinfection protocols, mechanical problems with dental unit components, contaminated sources of water, non-compliance of dental team members, and incompatibility of water treatment products [33]. However, this study identified a moderate positive association between dental clinics’ frequency of DUWL testing and their DUWL test failure rate. Put simply, clinics that elected to test their DUWLs more frequently were found to exhibit poorer quality water. We speculate that this paradoxical finding may have resulted from complacency in testing after receiving passing tests and increased concern regarding failed tests. Moreover, it is possible that clinics testing infrequently may have scheduled shock treatments immediately before sample collection, whereas those with standardized testing regimens may have provided more representative samples. Large clinics may have also been preferentially affected by factors such as the increased time and expense required to upkeep numerous dental units, rotating staff and clinicians, and variability in training and experience [36]. Nevertheless, this finding should be interpreted with caution. Future prospective studies should utilize questionnaires to assess clinicians’ knowledge and attitudes regarding DUWL care, as well as their current DUWL monitoring and maintenance protocols.

Fortunately, the number of Saskatchewan clinics engaging in external laboratory DUWL testing has grown with time. Despite a substantial decline in 2020, clinicians appear to be gaining awareness of the presence of microorganisms in procedural water. As well, a new Saskatchewan mandate took effect in 2019, requiring dental clinics to submit one yearly DUWL test to an external quality assurance laboratory [27]. Although a positive step, this regulation does not prescribe regular DUWL maintenance or follow-up actions in the event of a failed test. In recent times, the COVID-19 pandemic has placed a spotlight on IPAC principles and procedures [37]. With increased attention directed toward preventing nosocomial infections, this may be an excellent time for further regulatory efforts to improve the microbial quality of procedural water. Because DUWL flushing alone cannot maintain procedural water of acceptable quality [38, 39], the authors recommend daily use of evidence-based disinfectants [2, 20]. Interventions such as shock treatment with sodium hypochlorite should be required in the event of a failed DUWL test, followed by immediate retesting to confirm acceptable water quality. Periodic shock treatments may also eliminate microorganisms that develop resistance to the continuous exposure of low concentration biocides [19, 38]. Additionally, information on DUWL monitoring and maintenance should be implemented in both didactic and practical components of oral health program curricula. Such information can be reinforced in content from regulatory authorities and other professional organizations.

Although the present study examined the water quality and DUWL testing practices of numerous dental clinics over an 11-year period, it was limited by the constraints of retrospective research. Over time, changes in regulatory requirements, available evidence, and IPAC education may have influenced study outcomes. As well, each participating clinic joined the external laboratory registry at a different time, and self-selection bias may have occurred. Other limitations include the collection of water samples by dental teams rather than research personnel, the interpretation of DUWL tests using comparison charts, and the recording of data at the ordinal level. Moreover, although heterotrophic plate counts are a standard method for evaluating bacterial concentration, they cannot discern between pathogenic and innocuous organisms. Nevertheless, this study identified frequent microbial contamination of DUWLs in Saskatchewan dental clinics, along with infrequent and often insufficient follow-up efforts. To conclude, clinicians can ensure safe procedural water by implementing frequent DUWL monitoring, regular DUWL maintenance, evidence-based interventions when needed, and immediate retesting for verification.

## Data Availability

The datasets used and/or analyzed during the current study are available from the corresponding author on reasonable request.
